# Association between various insulin resistance surrogates and gallstone disease based on national health and nutrition examination survey

**DOI:** 10.1038/s41598-025-09482-1

**Published:** 2025-07-16

**Authors:** Rui Gong, Yuchen Ding, Kaiqi Yang, Xiaodie Meng, Xiujing Sun

**Affiliations:** Department of Gastroenterology, Beijing Friendship Hospital, National Clinical Research Center for Digestive Diseases, State Key Laboratory of Digestive Health, Beijing Digestive Disease Center, Beijing Key Laboratory for Precancerous Lesion of Digestive Diseases, Capital Medical University, Beijing, 100050 People’s Republic of China

**Keywords:** Insulin resistance surrogates, Gallstone disease, Metabolic syndrome, TyG index, NHANES, Diseases, Gastroenterology

## Abstract

Previous studies have reported potential associations between insulin resistance (IR) and the occurrence of gallstone disease (GSD). Recently, some novel indexes have been developed and applied for the assessment of IR. However, the association of these IR surrogates with GSD is unclear. The present study was designed to investigate the correlation between these novel IR surrogates and the risk of developing GSD and to explore the index with the best predictive value. We conducted a cross-sectional analysis using data from the National Health and Nutrition Examination Survey 2017-March 2020. Participants who self-reported a history of GSD and other necessary information were available and were enrolled. Ten IR surrogates were created based on previous literature. Univariate and multivariate logistic regression models were applied to estimate the effect of higher IR surrogates on the risk of GSD and restricted cubic splines (RCS) were used to show the dose-response relationships. Receiver operating characteristics (ROC) curves were depicted and the areas under the curves (AUC) were calculated to show the diagnostic value of ten indexes, respectively. Finally, the best index was selected and subgroup analysis was performed to further evaluate the risk level in different populations. Among the 2811 participants, 305 (11%) were diagnosed with GSD. According to results from univariate and multivariate logistic regression models, elevated levels of IR surrogates were significantly associated with an increased risk of GSD, including triglyceride glucose-body mass index (TyG-BMI), triglyceride glucose-waist circumference (TyG-WC), triglyceride glucose-waist to height ratio (TyG-WHtR), homeostasis model assessment-insulin resistance (HOMA-IR), metabolic score for insulin resistance (METS-IR), Chinese visceral adiposity index (CVAI), and lipid accumulation product (LAP). The RCS also confirmed the trend of positive correlation between the above indexes and GSD (*p* for overall < 0.0001). The ROC curves showed that TyG-WHtR demonstrated the strongest predictive power among these indexes, with an area under the curve of 0.6796 (95% CI 0.6513–0.7090). Subgroup analysis of the correlation between TyG-WHtR and GSD showed that the correlation was more pronounced among females, the never-married group, the nondiabetic group, and the group that did not use hypoglycemic or lipid-lowering medication. This study identified several novel IR surrogates that had significant positive correlations with the development of GSD. Among these indexes, TyG-WHtR was the strongest predictor of GSD, and the correlation was more pronounced in female groups and non-diabetic groups, which may provide significant value for screening, disease prediction, and early intervention in high-risk groups in clinical practice.

**Supplementary Information:**

The online version contains supplementary material available at 10.1038/s41598-025-09482-1.

## Introduction

Cholelithiasis or gallstone disease (GSD) is a worldwide prevalent disease, and ranks among the commonest gastrointestinal cause of hospital admission, especially in Western countries^1^. Although gallbladder stones are asymptomatic in the majority of cases, biliary colic may occur during an attack that warrants treatment. GSD also leads to serious complications such as acute cholecystitis, biliary pancreatitis, obstructive jaundice, cholangitis, and gallbladder cancer, where cholecystectomy as a preventive intervention may be required, imposing a substantial healthcare and financial burden^1,2^. Therefore, the detection of risk factors for the development of GSD is of paramount importance.

Increased cholesterol or decreased phospholipid and bile acid concentration in the bile and gallbladder hypomotility are required factors for GSD^3^. Previous studies showed pathogenetic pathways link cholesterol gallstones with widely diffused metabolic conditions which include insulin resistance (IR), obesity, metabolic syndrome, and type 2 diabetes (T2DM)^4,5^. Vitally, some studies indicate that the whole body and hepatic IR are associated with an increased risk of GSD, both in the general population and in high-risk groups (such as overweight and T2DM)^6–8^. IR and hyperinsulinemia increased hepatic cholesterol synthesis and excessive biliary cholesterol secretion, ultimately contributing to the increased risk of cholesterol stones and GSD^9,10^.

Up to now, hyperinsulinemic-euglycemic clamp (HEC) is considered the well-recognized gold standard for evaluating insulin sensitivity, but it is labor-intensive and time-consuming^11^. It has been found that IR usually presents as hyperglycemia, hyperinsulinemia, dyslipidemia, and central obesity, which can be evaluated by readily available laboratory and anthropometric parameters^12^. Consequently, various novel markers have been proposed as efficient surrogate indicators in recent years for measuring IR, including triglyceride-glucose index (TyG) and its derived indexes such as triglyceride glucose-body mass index (TyG-BMI), triglyceride glucose-waist circumference (TyG-WC), and triglyceride glucose-waist to height ratio (TyG-WHtR), homeostasis model assessment-insulin resistance (HOMA-IR), metabolic score for insulin resistance (METS-IR), triglyceride-to-high density lipoprotein cholesterol ratio (TG/HDL-C), visceral adiposity index (VAI), Chinese visceral adiposity index (CVAI), lipid accumulation product (LAP), and closely mirrors HEC in the assessment of IR^13–18^. Nevertheless, it remains obscure whether there exists an association between most IR surrogates mentioned above and the prevalence of GSD, with gaps in the comprehensive assessment and comparison among these surrogates. Therefore, this study aimed to evaluate the performance of various IR surrogates in the population with GSD and determine the optimal markers of the potential predictive value.

## Materials and methods

### Study population

NHANES is an ongoing nationally representative and cross-sectional survey, conducted by the Centers for Disease Control and Prevention, designed to assess the nutritional and health-related status and direct health promotion and disease prevention in the U.S. NHANES collected data via household interviews and standardized physical examinations implemented in the mobile examination center, releasing biannual, stratified, multistage probability samples of the non-institutionalized civilian U.S. population.

**Fig. 1:**
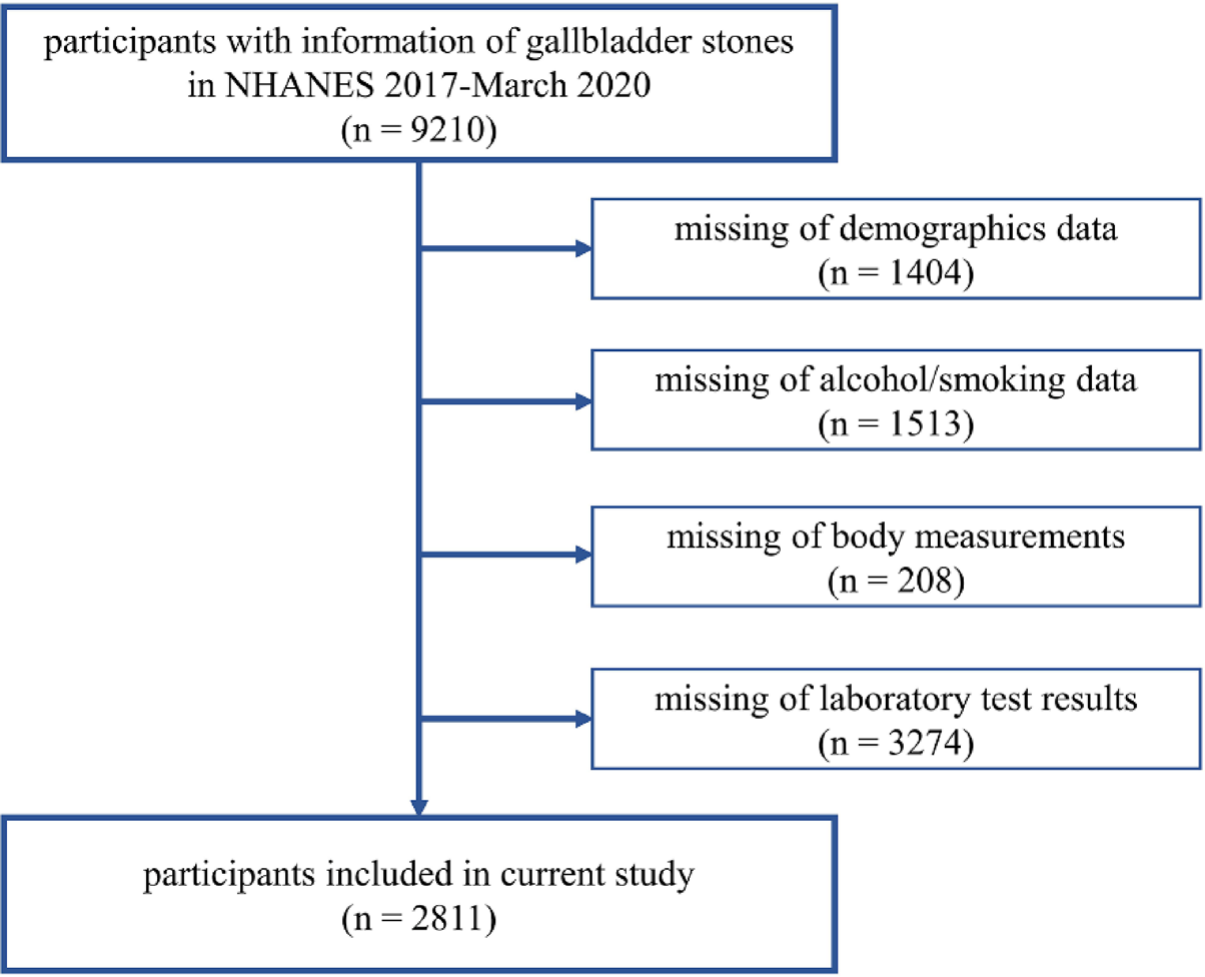
Number of participants included in/excluded from the study and reasons for exclusion.

The population of this research was drawn from NHANES 2017-March 2020 Pre-Pandemic database, in which a total of 15,560 people participated in the project. Participants aged 20 or older who specifically self-reported GSD-related information on the medical condition questionnaire were enrolled. Initially, participants with GSD-related information were included in the study. However, individuals were subsequently excluded if they lacked the necessary data for calculating IR surrogate indicators or if they had missing data on baseline covariates. Finally, 2811 records were included in this study. The flow diagram is shown in Fig. 1. The NHANES is approved by the National Center for Health Statistics Ethics Review Board. All participants provided written informed consent.

### Definitions of gallstone disease and IR surrogate indicators

The presence of GSD was recorded as an outcome variable. If the participant answered ‘Yes’ to the question 'Has a doctor or other health professional ever told you that you had GSDs? ', we defined him as having a history of GSDs. IR surrogate indicators were calculated as described previously, and the formulas were listed in Table 1.

**Table 1:** IR surrogates and corresponding formulas.

IR surrogates	Formulas	References
TyG	Ln [TG (mg/dL) × FBG (mg/dL)/2]	^19^
TyG-BMI	TyG × Height (m)/[Weight (kg)]^2^	^20^
TyG-WC	TyG × WC (cm)	^20^
TyG-WHtR	TyG × WC (cm)/Height (m)	^21^
HOMA-IR	FBG (mmol/L) × FINS (μU/ml)/22.5	^22^
METS-IR	(Ln (2 × FBG (mg/dL)) + TG (mg/dL)) × BMI/Ln (HDL-C (mg/dL))	^23^
TG/HDL-C	TG (mg/dL)/HDL-C (mg/dL)	^24^
VAI	Male: [WC (cm)/(39.68 + 1.88 × BMI)] × [TG (mmol/L)/1.03] × [1.31/HDL-C (mmol/L)]	^25^
	Female: [WC (cm)/(36.58 + 1.89 × BMI)] × [TG (mmol/L)/0.81] × [1.52/HDL-C (mmol/L)]	
CVAI	Male: − 267.93 + 0.68 × age + 0.03 × BMI + 4.00 × WC (cm) + 22.00 × Lg [TG (mmol/L)] − 16.32 × HDL-C (mmol/L)	^26^
	Female: − 187.32 + 1.71 × age + 4.32 × BMI + 1.12 × WC (cm) + 39.76 × Lg [TG (mmol/L)] − 11.66 × HDL-C (mmol/L)	
LAP	Male: [WC (cm) − 65] × [fasting TG (mmol/L)]	^27^
	Female: [WC (cm) − 58] × [fasting TG (mmol/L)]	

### Covariates

Covariates were chosen based on clinical judgment and previous studies^28,29^. Sociodemographic characteristics included sex (male or female), age (years), race (Mexican American, non-Hispanic Black, non-Hispanic White, other Hispanic, or other races), marital status (married, divorced or never married), education attainment (above high school, high school, or below high school), and income level, which was measured by poverty-to-income ratio (PIR) and categorized into three brackets (low-income: < 1.30, middle-income: 1.30–3.50, or high-income: > 3.5)^30^. Behavioral risk factors included smoking history (yes or no), alcohol consumption (above once a month, once a month or less, or never), and physical activity, evaluated by metabolic equivalent (MET) scores and classified as no physical activity (0 MET min/week), low-level physical activity (1–599 MET-min/ week), moderate-level physical activity (600–1200 MET min/week), and high-level physical activity (> 1200 MET min/week)^31^. Comorbidities included diabetes and hypertension. Diabetes was defined by self-reported history of diabetes and glucose-lowering medication use and the results of fasting blood glucose and hemoglobin A1c (FBG > 6.9 mmol/L and/or hemoglobin A1c > 6.5%). Hypertension was defined by self-reported history of hypertension and antihypertensive medication use and blood pressure measurements in mobile examination centers (≥ 140/90 mmHg). Finally, the anthropometric parameters including body mass index (BMI), height, weight, waist circumference (WC), and laboratory information such as blood glucose and lipid profiles were collected (details shown in Table 2).

**Table 2:** Baseline characteristics of the study population.

Characteristic	Overall (2811)	GSD		*P*-value
		No (2506; 89%)	Yes (305; 11%)	
Gender				< 0.001
Male	1423.00 (49.12%)	1339.00 (52.47%)	84.00 (23.20%)	
Female	1388.00 (50.88%)	1167.00 (47.53%)	221.00 (76.80%)	
Age (years)	47.00 (33.00, 61.00)	46.00 (32.00, 60.00)	56.00 (46.00, 66.00)	< 0.001
Race				0.074
Mexican American	354.00 (8.75%)	306.00 (8.89%)	48.00 (7.66%)	
Non-Hispanic Black	699.00 (10.78%)	653.00 (11.59%)	46.00 (4.51%)	
Non-Hispanic White	1059.00 (65.40%)	927.00 (64.57%)	132.00 (71.81%)	
Other Hispanic	268.00 (6.24%)	230.00 (6.18%)	38.00 (6.74%)	
Other/multiracial	431.00 (8.83%)	390.00 (8.77%)	41.00 (9.28%)	
Marital status				0.027
Divorced	617.00 (19.43%)	531.00 (18.64%)	86.00 (25.53%)	
Married	1680.00 (62.09%)	1496.00 (62.07%)	184.00 (62.22%)	
Never married	514.00 (18.48%)	479.00 (19.28%)	35.00 (12.25%)	
Level of PIR				0.10
High	959.00 (48.31%)	871.00 (49.40%)	88.00 (39.85%)	
Medium	1123.00 (34.10%)	979.00 (32.89%)	144.00 (43.48%)	
Low	729.00 (17.59%)	656.00 (17.71%)	73.00 (16.67%)	
Educational level				0.3
Above high school	1692.00 (65.28%)	1523.00 (65.92%)	169.00 (60.34%)	
High school	669.00 (25.55%)	592.00 (24.87%)	77.00 (30.82%)	
Below high school	450.00 (9.17%)	391.00 (9.21%)	59.00 (8.84%)	
Diabetes	632.00 (16.04%)	514.00 (14.52%)	118.00 (27.79%)	< 0.001
Hypertension	1267.00 (38.80%)	1089.00 (36.54%)	178.00 (56.29%)	< 0.001
Frequency of alcohol				< 0.001
Never	608.00 (16.96%)	505.00 (15.24%)	103.00 (30.30%)	
Once a month or less	963.00 (32.91%)	854.00 (32.90%)	109.00 (32.94%)	
Above once a month	1240.00 (50.13%)	1147.00 (51.85%)	93.00 (36.76%)	
History of smoking	1315.00 (45.73%)	1161.00 (44.89%)	154.00 (52.22%)	0.060
Hypoglycemic agents	423.00 (10.56%)	346.00 (9.46%)	77.00 (19.09%)	< 0.001
Lipid-lowering drugs	689.00 (21.97%)	583.00 (20.94%)	106.00 (29.96%)	0.018
Physical activity level				0.2
No physical activity	664.00 (18.56%)	568.00 (17.87%)	96.00 (23.96%)	
Low level physical activity	327.00 (10.96%)	288.00 (10.60%)	39.00 (13.70%)	
Moderate level physical activity	310.00 (12.54%)	285.00 (12.86%)	25.00 (10.09%)	
High level physical activity	1510.00 (57.94%)	1365.00 (58.67%)	145.00 (52.26%)	
BMI (kg/m^2^)	28.60 (24.70, 33.50)	28.20 (24.40, 33.00)	31.23 (27.00, 38.10)	< 0.001
Height (cm)	168.40 (161.50, 175.60)	169.00 (162.00, 176.10)	165.20 (158.45, 169.74)	< 0.001
Weight (kg)	82.70 (69.40, 97.00)	82.10 (69.15, 96.58)	85.88 (72.77, 101.74)	0.033
Waist circumference (cm)	99.10 (88.30, 111.10)	98.20 (87.40, 110.50)	106.31 (95.53, 117.40)	< 0.001
TG (mg/dl)	88.00 (61.00, 133.00)	87.87 (59.75, 132.00)	102.00 (67.00, 147.81)	0.040
FBG (mg/dl)	102.00 (95.00, 111.00)	102.00 (95.00, 110.00)	105.00 (99.00, 119.00)	< 0.001
TC (mg/dl)	180.00 (157.00, 208.00)	180.00 (157.00, 207.07)	181.00 (155.95, 213.58)	0.6
LDL-C (mg/dl)	107.00 (85.00, 131.00)	107.00 (86.00, 130.00)	106.53 (82.00, 131.00)	> 0.9
HDL-C (mg/dl)	51.00 (42.00, 63.00)	51.00 (42.00, 63.00)	52.00 (43.00, 63.00)	0.8
TyG	8.45 (8.00, 8.90)	8.43 (7.99, 8.86)	8.65 (8.16, 9.01)	0.004
TyG-BMI	243.11 (202.88, 292.65)	239.55 (199.93, 287.44)	268.49 (230.05, 325.02)	< 0.001
TyG-WC	848.50 (722.09, 973.22)	836.39 (711.76, 959.30)	925.13 (788.33, 1,058.77)	< 0.001
TyG-WHtR	5.03 (4.28, 5.75)	4.96 (4.22, 5.65)	5.53 (4.90, 6.38)	< 0.001
HOMA-IR	2.39 (1.42, 4.26)	2.25 (1.37, 4.17)	3.47 (1.98, 5.87)	< 0.001
METS-IR	42.14 (34.37, 50.99)	41.66 (33.91, 50.15)	45.85 (38.14, 56.64)	< 0.001
TG/HDL-C	1.71 (1.02, 2.89)	1.70 (1.00, 2.84)	1.87 (1.12, 3.11)	0.093
VAI	1.24 (0.73, 2.16)	1.21 (0.70, 2.10)	1.51 (0.96, 2.72)	< 0.001
CVAI	136.10 (76.57)	132.05 (76.81)	167.46 (67.01)	< 0.001
LAP	38.49 (20.53, 67.89)	36.32 (19.25, 64.65)	56.32 (29.72, 81.25)	< 0.001

### Statistical analysis

According to the analytic guideline of the NHANES, fasting subsample weights were constructed for analysis as appropriate to obtain national estimates representative of the U.S. civilian non-institutionalized population.

The normality of continuous variables was comprehensively assessed using the Kolmogorov–Smirnov test, P-P plots, Q-Q plots, and frequency histograms. Continuous variables were presented as mean and standard deviation (for normal distribution), or median and interquartile ranges (for skewed distribution), compared by t-test or Mann–Whitney test, respectively. Categorical variables were presented as percentages and compared by Chi-square test. Baseline characteristics were compared according to suffering from GSD or not, and variables were described in weighted forms. All IR surrogates were categorized into quartiles, and the proportion of GSDs was compared between groups with different quartiles of IR surrogates. Weighted receiver operating characteristic (ROC) curves were depicted for each IR surrogate predicting the presence of GSD, and the area under the curve (AUC) was calculated to evaluate their performance. Multivariate logistic regression analysis was performed to calculate the odds ratios (OR) and 95% confidence intervals (CI) for GSD in the comparison of the highest quartile to the lowest quartile. The multivariate logistic regression model was adjusted for confounders including age, sex, race, marital status, education attainment, income level, smoking history, alcohol consumption, physical activity, and the comorbidity of diabetes and hypertension. Restricted cubic splines (RCS) were conducted to illustrate the relationships between IR surrogate indicators and GSD. A likelihood ratio test compared the RCS model to a linear model to test for nonlinearity. Subgroup analyses were further performed to separately evaluate the associations across populations with or without diabetes.

Statistical significance was determined by a two-tailed *P*-value ≤ 0.05. Statistical analyses were conducted using SPSS (v. 26.0) and R (v. 4.2.2).

## Results

### Baseline characteristics

Characteristics of the study population are summarized in Table 2. Amongst a total of 2811 (total weighted n = 184,384,451) participants involved, 305 (11%) records were identified as suffering from GSD. Participants with GSD tended to be older and showed higher proportions of females, divorced, diabetes, hypertension, and drug management of glucose and lipid, and showed a lower proportion of high-frequency drinking (all *p* < 0.05). As for body measurements and laboratory indicators, participants with GSD had higher BMI, WC, fasting blood glucose (FBG), triglyceride (TG), and lower height (all *p* < 0.05). In terms of surrogate indicators, the levels of TyG, TyG-BMI, TyG-WC, TyG-WHtR, HOMA-IR, METS-IR, VAI, CVAI, and LAP were all significantly higher in the population with GSD (all *p* < 0.05), except for TG/HDL-C.

### Predictive value of IR surrogate indicators

**Fig. 2:**
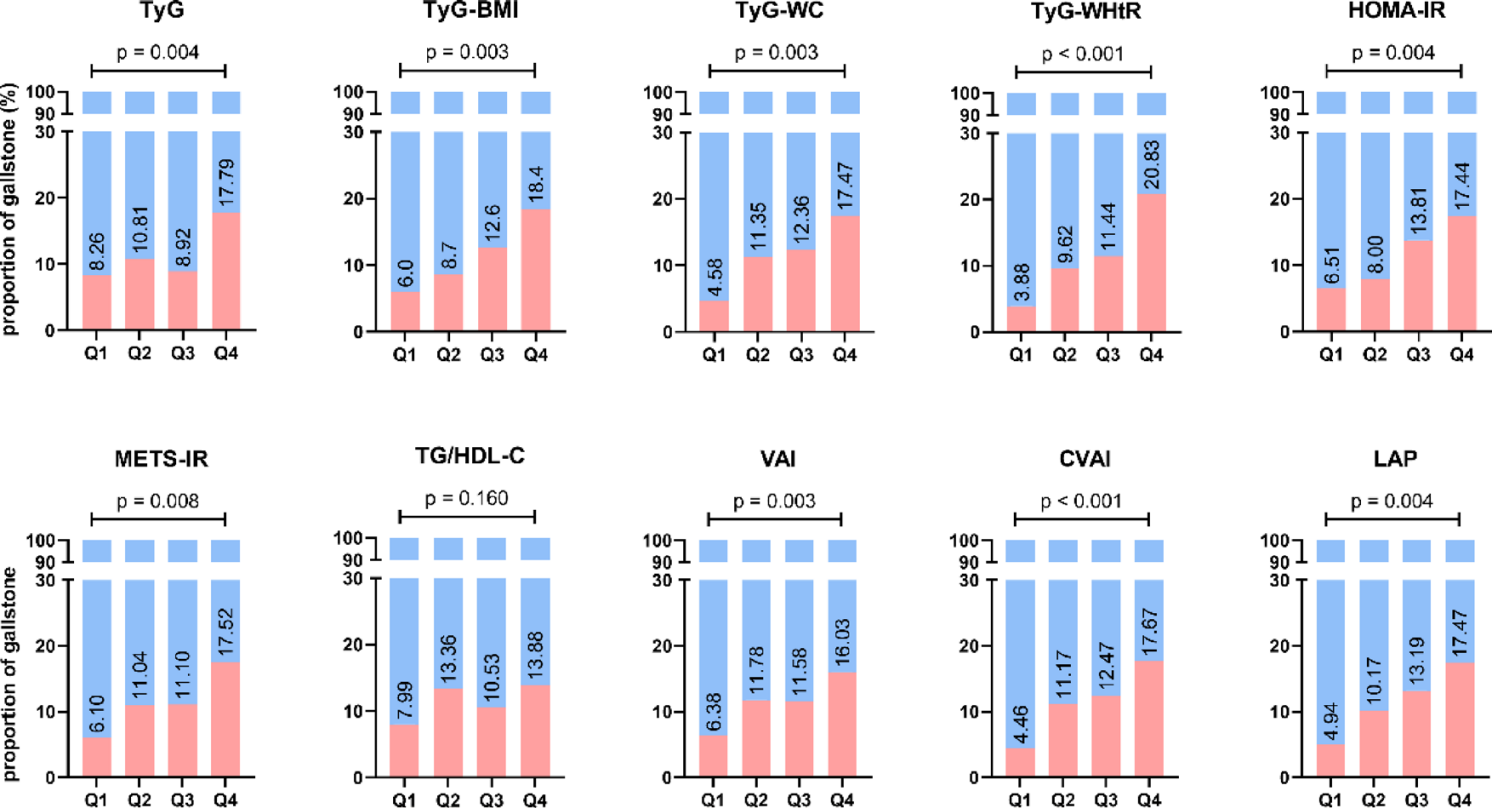
Frequency of gallstone among groups with different quartiles of insulin resistance surrogates.

As shown in Fig. 2, there were significant differences in the prevalence of GSD among groups with different IR surrogate indicator quartiles of TyG, TyG-BMI, TyG-WC, TyG-WHtR, HOMA-IR, METS-IR, VAI, CVAI and LAP (all *p* < 0.05). Apparently, step-like increases of GSD incidence were presented with the increasing level quartiles of TyG-BMI, TyG-WC, TyG-WHtR, HOMA-IR, METS-IR, CVAI and LAP.

Each IR surrogate indicator was utilized as both a continuous variable and quartile to explore the associations with GSD (Table 3 and Supplementary Table). Weighted unadjusted and fully adjusted logistic regression models showed statistically significant positive correlations between TyG-BMI (4^th^ vs 1^st^ quartile, adjusted OR 2.907, 95% CI 1.254–6.737, *p* = 0.022), TyG-WC (4^th^ vs 1^st^ quartile, adjusted OR 3.730, 95% CI 1.436–9.685, *p* = 0.016), TyG-WHtR (4^th^ vs 1^st^ quartile, adjusted OR 4.026, 95% CI 1.314–12.34, *p* = 0.024), HOMA-IR (4^th^ vs 1^st^ quartile, adjusted OR 3.072, 95% CI 1.431–6.598, *p* = 0.013), HOMA-IR (4^th^ vs 1^st^ quartile, adjusted OR 3.403, 95% CI 1.528–7.580, *p* = 0.011) and CVAI (4^th^ vs 1^st^ quartile, adjusted OR 3.988, 95% CI 1.410–11.28, *p* = 0.019) levels and GSD prevalence. As the results of unadjusted and adjusted logistic regression models showed, TyG-WHtR, with the highest OR and 95% CI, was exhibited to be the strongest risk predictor for GSD.

**Table 3:** Predictive value of each insulin resistance (IR) surrogates for gallstone disease risk.

IR surrogates	Variate type	Unadjusted analysis		Adjusted analysis*	
		OR (95% CI)	*P*-value	OR (95% CI)	*P*-value
TyG	Q4 vs. Q1	2.403 (1.404, 4.114)	0.003	1.596 (0.723, 3.524)	0.189
	Continuous	1.488 (1.142, 1.939)	0.002	1.197 (0.798, 1.794)	0.294
TyG-BMI	Q4 vs. Q1	3.524 (1.872, 6.635)	< 0.001	2.907 (1.254, 6.737)	0.022
	Continuous	1.006 (1.004, 1.008)	< 0.001	1.006 (1.003, 1.008)	< 0.001
TyG-WC	Q4 vs. Q1	4.408 (2.260, 8.597)	< 0.001	3.730 (1.436, 9.685)	0.016
	Continuous	1.003 (1.002, 1.003)	< 0.001	1.002 (1.001, 1.004)	< 0.001
TyG-WHtR	Q4 vs. Q1	6.519 (2.829, 15.02)	< 0.001	4.026 (1.314, 12.34)	0.024
	Continuous	1.700 (1.477, 1.958)	< 0.001	1.451 (1.183, 1.778)	< 0.001
HOMA − IR	Q4 vs. Q1	3.032 (1.894, 4.855)	< 0.001	3.072 (1.431, 6.598)	0.013
	ln (HOMA − IR)	1.692 (1.474, 1.942)	< 0.001	1.819 (1.401, 2.362)	< 0.001
METS − IR	Q4 vs. Q1	3.268 (1.784, 5.985)	< 0.001	3.403 (1.528, 7.580)	0.011
	Continuous	1.030 (1.020, 1.041)	< 0.001	1.031 (1.018, 1.044)	< 0.001
TG/HDL-C	Q4 vs. Q1	1.855 (1.115, 3.085)	0.020	1.602 (0.846, 3.033)	0.116
	ln (TG/HDL-C)	1.211 (0.965, 1.520)	0.083	1.161 (0.852, 1.583)	0.254
VAI	Q4 vs. Q1	2.801 (1.629, 4.818)	< 0.001	1.563 (0.706, 3.464)	0.208
	ln (VAI)	1.562 (1.251, 1.951)	< 0.001	1.187 (0.877, 1.607)	0.180
CVAI	Q4 vs. Q1	4.599 (2.276, 9.294)	< 0.001	3.988 (1.410, 11.28)	0.019
	Continuous	1.006 (1.004, 1.008)	< 0.001	1.006 (1.003, 1.009)	< 0.001
LAP	Q4 vs. Q1	4.073 (2.016, 8.228)	< 0.001	2.592 (0.935, 7.187)	0.062
	ln (LAP + 1)	1.850 (1.490, 2.297)	< 0.001	1.544 (1.145, 2.083)	< 0.001

*Adjusted for age, sex, race, marital status, income level, diabetes, hypertension, alcohol frequency, smoking history, glucose-lowering medication taking, and lipid-lowering medication taking.

**Fig. 3:**
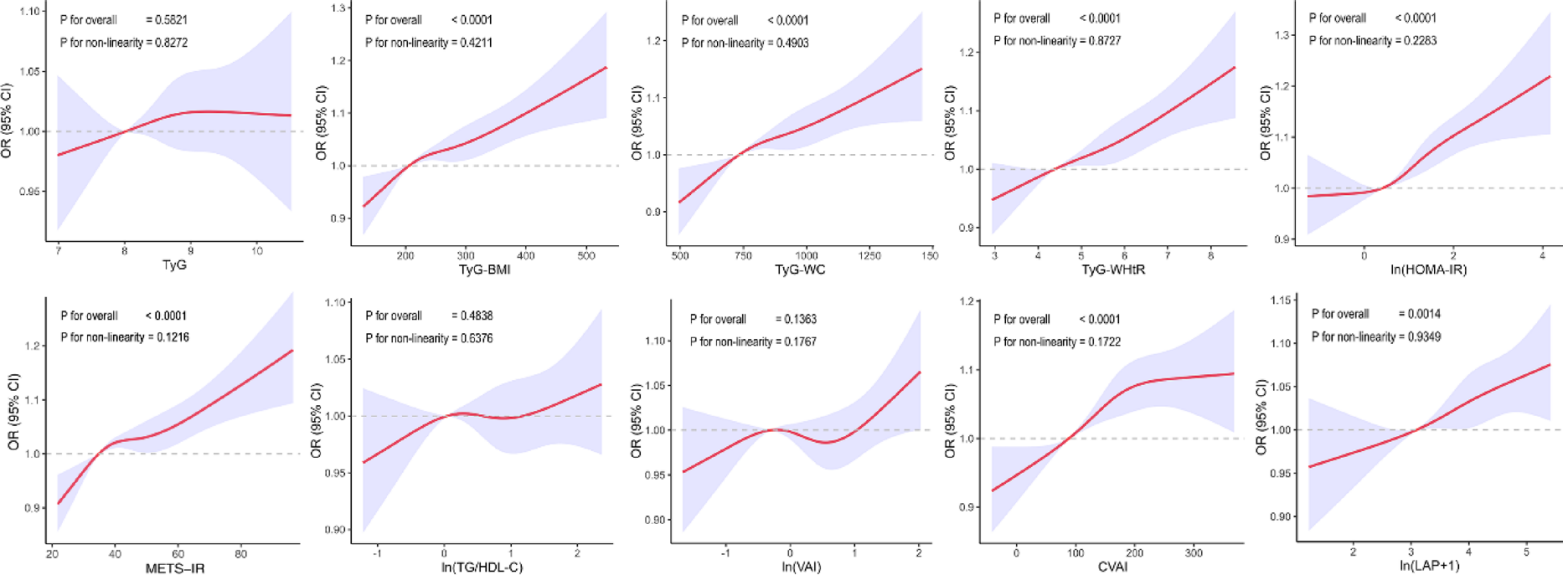
Restricted cubic spine models of the relationships between insulin resistance surrogates and gallstone disease risk. Adjusted for age, sex, race, marital status, income level, diabetes, hypertension, alcohol frequency, smoking history, glucose-lowering medication taking, and lipid-lowering medication taking.

Additionally, multivariate-adjusted RCS were employed and elucidated the significant links between the level of TyG-BMI, TyG-WC, TyG-WHtR, HOMA-IR, METS-IR, CVAI, and LAP and the risk of GSD occurrence (all P-overall < 0.0001, Fig. 3). No non-linear relationship was examined.

**Fig. 4:**
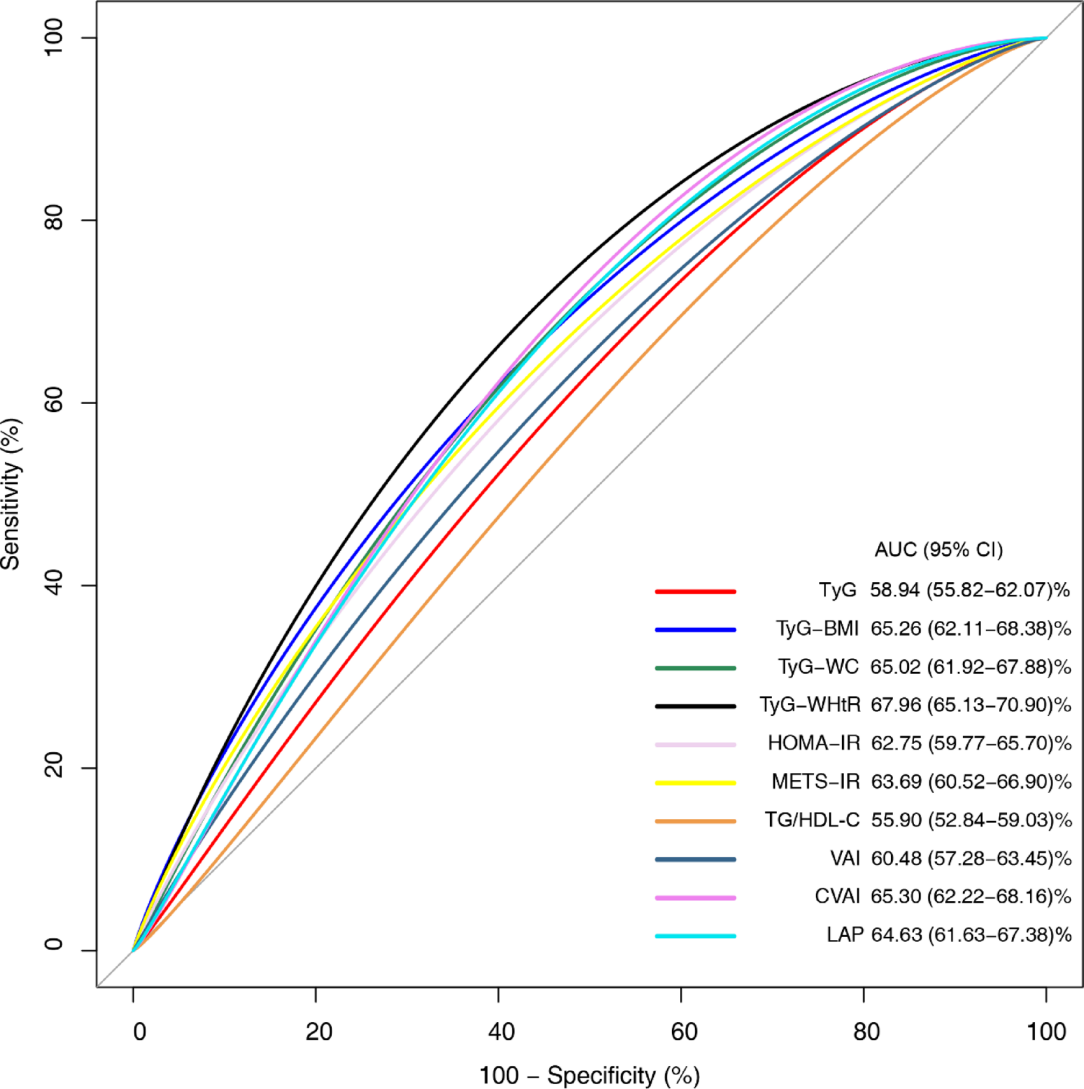
Receiver operating characteristic of insulin resistance surrogate indexes for gallstone disease.

Based on the results of ROC curves, TyG-WHtR exhibited the strongest diagnostic ability for GSD manifested as the maximum AUC of 0.6796 (Fig. 4).

### Subgroup analyses

**Fig. 5:**
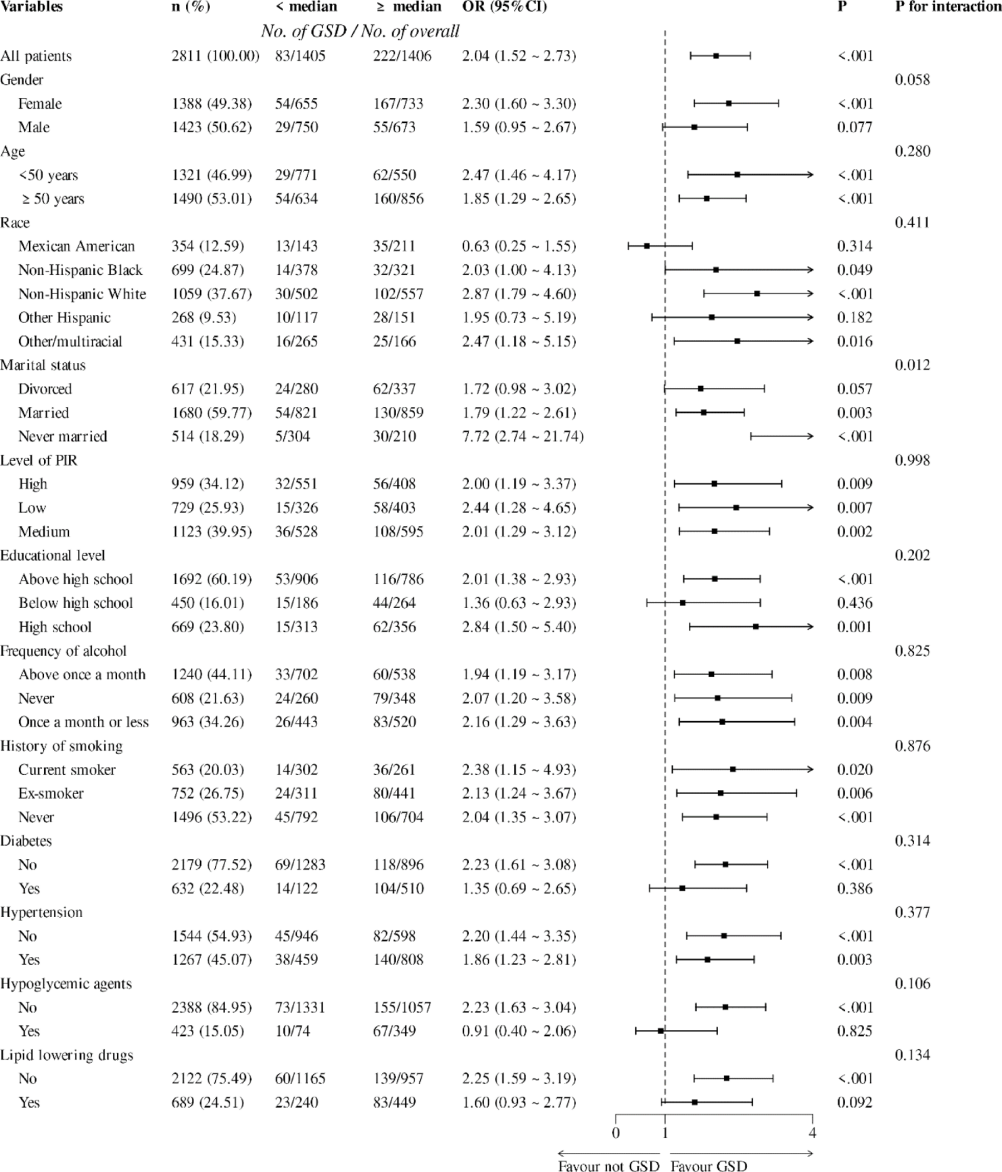
Subgroup analysis of the association between triglyceride glucose-waist to height ratio and gallstone disease in different populations. Adjusted for age, sex, race, marital status, income and education level, hypertension, alcohol frequency, smoking history, and hypoglycemic and lipid-lowering medication taking.

As shown in Fig. 5, the study further conducted subgroup analyses of the association between TyG-WHtR and GSD in populations of different genders, ages, races, marital statuses, income levels, education levels, smoking and drinking histories, comorbidities, and medication use statuses. Subgroup analyses after adjusting for confounders showed that TyG-WHtR levels were significantly and positively associated with the development of GSD in the female population (OR 2.30, 95% CI 1.60–3.30, *p* < 0.001). In terms of race, the positive correlation between TyG-WHtR and the development of GSD was most significant among non-Hispanic whites (OR 2.87, 95% CI 1.79–4.60, *p* < 0.001). As for marital status, the positive correlation between TyG-WHtR and GSD occurrence was most significant in never married population (OR 7.72, 95% CI 2.74–21.74, *p* < 0.001) and there was an interaction (p for interaction = 0.012). In terms of disease history, TyG-WHtR was significantly and positively associated with the development of GSD in the non-diabetic population relative to the diabetic group (OR 2.23, 95% CI 1.61–3.08, *p* < 0.001). In terms of medication use, the positive association of TyG-WHtR with the development of GSD was statistically significant in those who did not use hypoglycemic agents and those who did not use lipid-lowering drugs (OR 2.23, 95% CI 1.63–3.04, *p* < 0.001; OR 2.25, 95% CI 1.59–3.59, *p* < 0.001).

## Discussion

In the current study, we revealed a strong positive association between IR and GSD regardless of the presence or absence of diabetes, and TyG-WHtR was confirmed to display superiority over other IR surrogate indicators on risk prediction and diagnostic value for GSD.

IR is defined as a pathological condition characterized by the decreased sensitivity of insulin target organs (adipose tissue, skeletal muscle, liver) to the biological effects of insulin^32^. Overweight and obesity resulting from unhealthy diet, sedentary lifestyle, smoking, and environmental contaminants are the most important acquired risk factors^33,34^. IR leads to a cluster of disorders including diabetes and impaired FBG, hyperinsulinemia, obesity (especially abdominal obesity), hypertension, and dyslipidemia^33^.

Patients with IR manifest higher plasma insulin levels^35^. Scragg et al. were among the first to discover the relationship between increased plasma insulin levels and increased risk of gallstones^36^. The association is probably mediated through complicated and diverse etiologies. Metabolic syndrome is a collection of metabolic abnormalities, including obesity, hypertension, dyslipidemia, and hyperglycemia^37^, and IR serves as the core of metabolic syndrome. Diabetes is closely associated with aberrations in lipid metabolism and leads to the development of hyperlipidemia and hypercholesterolemia. Additionally, patients with diabetes are frequently accompanied by dysautonomia, resulting in poor gallbladder contraction function and cholestasis^38^. Stiffening of the gallbladder wall resulting from the cross-linking of matrix produced by carbohydrate metabolism also plays a role^39^. Nevertheless, even in the absence of diabetes, hyperinsulinemia can promote cholesterol synthesis by increasing the hydroxymethylglutaryl-coenzyme A reductase activity and enhancing the amount of low-density lipoprotein cholesterol (LDL-C) transferred to the liver by improving the activity of LDL-C receptors, both contributing to biliary cholesterol supersaturation and consequently promoting the formation of gallstones^9,40^. Moreover, the elevation of insulin can inhibit the key enzyme cholesterol-7α-hydroxylase of bile salt synthesis. Changes in bile acid composition and increased nucleation of bile accelerate the cholesterol crystallization in bile, thereby promoting the formation of gallstones^41,42^. Obesity, especially visceral obesity, often indicating visceral fat obesity, tends to cause gallbladder dysmotility due to reduced sensitivity to cholecystokinin^43^. However, IR leading to gallbladder dysmotility also been found in lean nondiabetic subjects^39^. This is perhaps associated with hyperinsulinemia triggering the sodium–potassium pump to imbalance the internal environment of the gallbladder muscle cells, leading to abnormal gallbladder emptying.

The operational complexity and time consumption of HEC restricts its broad clinical application. HOMA-IR was first proposed in 1985 and developed as the most common method due to its ease of practical application and most widely used^14,44^. Later, the TyG index was developed and considered as a cost-effective indicator of IR, calculated from common laboratory parameters. HOMA-IR and elevated TyG index values coincide with an augmented likelihood of gallstone occurrence, especially in females^38,45,46^. These years, an increasing number of studies on the influence of the TyG-derived indicators including TyG-BMI, TyG-WC, and TyG-WHtR. METS-IR is also an important alternative index of IR, which has been reported to be associated with cardiovascular mortality^18^, hypertension^23^, and kidney stones^47^. More recently, novel IR surrogates including VAI, CVAI, LAP, and TG/HDL-C have demonstrated clinical effectiveness in the prediction of cardiovascular disease, cerebrovascular disease, metabolic syndrome, and chronic kidney disease^12,48–50^, but no studies have demonstrated the association with GSD so far. In our study, CVAI and LAP were found to elicit a statistically significant increase in GSD risk. The most powerful IR predictor of GSD was proved to be TyG-WHtR. Previous study has shown that fasting TG mainly reflects IR from the adipose tissue, whereas FBG mainly reflects IR from the liver^51^. It was suggested that the waist-to-height ratio is a better alternative for assessing abdominal obesity compared to other anthropometric indicators^52^. The waist-to-height ratio reflects visceral obesity, which links closely to IR and may decrease gallbladder peristalsis^39^. Therefore, TyG-WHtR, calculated by TG, FBG, and waist-to-height ratio, may promote the formation of gallstones from the above different dimensions.

As the findings of the present study, many previous studies have shown that gallstones are more common in women, which may be associated with elevated fasting insulin levels due to estrogen^53,54^. In agreement with Wang et al., we also found a positive correlation between TyG-WHtR and GSD in all age groups^29^. Interestingly, the results of this study suggest that the promotion of GSD by TyG-WHtR was more pronounced in never-married populations, which may be associated with the fact that unmarried populations are more inclined to irregular dietary habits, such as frequent eating out, high-fat and high-calorie diets, or dietary irregularities, and may lack family support, which may lead to insufficient health management such as lack of physical activity and neglect of regular medical checkups. In several previous studies exploring the correlation between IR surrogates and cardiovascular disease, positive correlations have been found only in diabetic populations^55–57^. However, this study found no significant correlation between TyG-WHtR and GSD in the subgroup of diabetic patients, but instead, positive results were found in the non-diabetic population, which is also in line with the findings of Gong et al.^58^, who did not find a correlation between TyG and GSD in diabetic patients. This suggests that the relationship between IR surrogates and various diseases may vary in different populations. Patients with diabetes usually take medications to control blood glucose levels, such as insulin, biguanides, or sulfonylureas, which not only directly affect glycemic regulation, but may also affect lipid levels^59,60^. Therefore, even in the presence of IR, these drugs may indirectly affect the calculation of TyG-WHtR by regulating blood glucose and lipid levels, thus masking the true link between TyG-WHtR and GSD in diabetic patients. This may be one of the reasons why there is no correlation between TyG-WHtR and GSD in diabetic patients. Similarly, the results of the correlation between TyG-WHtR and GSD found only in the population without the application of hypoglycemic and lipid-lowering drugs may also be due to the above-mentioned reasons.

This is the first study comprehensively exploring the superiority of various IR surrogates on risk prediction for GSD. Our study exhibits several strengths. The comprehensive analysis covered all IR surrogates considered reliable in previous studies. And the robust methodology guarantees the correctness of the optimal index and its applicability to the overall U.S. population. There are however also limitations in our study. First, its cross-sectional design precludes causal determination. Secondly, the determination of GSD was derived from self-report questionnaires and could be susceptible to reporting or recall bias. Thirdly, the findings from NHANES data, based on the U.S. population, may not be applicable to other populations with different races, lifestyles, dietary habits, and obesity rates. Expanding research to diverse populations utilizing well‐designed randomized controlled studies will enhance the applicability of our results.

## Conclusions

In conclusion, this study utilized data from a cross-sectional study conducted in the U.S. to determine significant correlations between several IR surrogates and GSD. Among these surrogates, TyG-WHtR was the strongest predictor of GSD, and the correlation was more pronounced in the female and nondiabetic populations, which may provide significant value for screening high-risk populations, disease prediction, and early intervention in clinical practice.

## Electronic supplementary material

Below is the link to the electronic supplementary material.

Supplementary Material 1

## Data Availability

The data that support the findings of this study are openly available in public databases NHANES. Further inquiries can be directed to the corresponding authors.

## Abbreviations

NHANES: National health and nutrition examination survey
GSD: Gallstone disease
IR: Insulin resistance
T2DM: Type 2 diabetes
HEC: Hyperinsulinemic-euglycemic clamp
TyG: Triglyceride-glucose index
TyG-BMI: Triglyceride glucose-body mass index
TyG-WC: Triglyceride glucose-waist circumference
TyG-WHtR: Triglyceride glucose-waist-to-height ratio
TG/HDL-C: Triglyceride-to-high density lipoprotein cholesterol ratio
HOMA-IR: Homeostasis model assessment-insulin resistance
METS-IR: Metabolic score for insulin resistance
VAI: Visceral adiposity index
CVAI: Chinese visceral adiposity index
LAP: Lipid accumulation product
PIR: Poverty-to-income ratio
MET: Metabolic equivalent
BMI: Body mass index
WC: Waist circumference
ROC: Receiver operating characteristic curve
AUC: Area under the curve
OR: Odds ratios
CI: Confidence intervals
RCS: Restricted cubic spline
FBG: Fasting blood glucose
TG: Triglyceride
LDL-C: Low-density lipoprotein cholesterol
