# Supplementary material for: Association between various insulin resistance surrogates and gallstone disease based on national health and nutrition examination survey
Source: Sci Rep. 2025 Jul 16;15:25877. doi: 10.1038/s41598-025-09482-1 (PMC12267482; doi:10.1038/s41598-025-09482-1)
Supplement: Supplementary file 1 — Supplementary Material 1 [file 41598_2025_9482_MOESM1_ESM.docx]

**Supplementary Table** Predictive value of each insulin resistance (IR) surrogates in each quartile subgroups for gallstone disease risk.

| IR surrogates | Quartiles | Unadjusted analysis | | Adjusted analysis* | |
| --- | --- | --- | --- | --- | --- |
|  |  | OR (95% CI) | P-value | OR (95% CI) | P-value |
| TyG | Q1 | Reference |  | Reference |  |
|  | Q2 | 1.347 (0.723, 2.509) | 0.332 | 1.080 (0.487, 2.392) | 0.814 |
|  | Q3 | 1.088 (0.510, 2.320) | 0.820 | 0.761 (0.298, 1.943) | 0.487 |
|  | Q4 | 2.403 (1.404, 4.114) | 0.003 | 1.596 (0.723, 3.524) | 0.189 |
| TyG-BMI | Q1 | Reference |  | Reference |  |
|  | Q2 | 1.483 (0.598, 3.678) | 0.378 | 1.280 (0.410, 4.000) | 0.602 |
|  | Q3 | 2.252 (1.077, 4.708) | 0.032 | 2.121 (0.793, 5.678) | 0.107 |
|  | Q4 | 3.524 (1.872, 6.635) | <0.001 | 2.907 (1.254, 6.737) | 0.022 |
| TyG-WC | Q1 | Reference |  | Reference |  |
|  | Q2 | 2.666 (1.088, 6.528) | 0.033 | 2.433 (0.808, 7.325) | 0.093 |
|  | Q3 | 2.938 (1.304, 6.619) | 0.012 | 3.013 (1.002, 9.059) | 0.050 |
|  | Q4 | 4.408 (2.260, 8.597) | <0.001 | 3.730 (1.436, 9.685) | 0.016 |
| TyG-WHtR | Q1 | Reference |  | Reference |  |
|  | Q2 | 2.636 (0.894, 7.778) | 0.077 | 2.220 (0.574, 8.585) | 0.190 |
|  | Q3 | 3.199 (1.130, 9.056) | 0.030 | 2.695 (0.683, 10.63) | 0.123 |
|  | Q4 | 6.519 (2.829, 15.02) | <0.001 | 4.026 (1.314, 12.34) | 0.024 |
| HOMA−IR | Q1 | Reference |  | Reference |  |
|  | Q2 | 1.249 (0.593, 2.629) | 0.542 | 1.186 (0.481, 2.923) | 0.647 |
|  | Q3 | 2.300 (1.027, 5.154) | 0.044 | 2.123 (0.781, 5.774) | 0.111 |
|  | Q4 | 3.032 (1.894, 4.855) | <0.001 | 3.072 (1.431, 6.598) | 0.013 |
| METS−IR | Q1 | Reference |  | Reference |  |
|  | Q2 | 1.910 (0.796, 4.583) | 0.139 | 2.062 (0.699, 6.079) | 0.146 |
|  | Q3 | 1.921 (0.895, 4.124) | 0.090 | 2.235 (0.801, 6.237) | 0.100 |
|  | Q4 | 3.268 (1.784, 5.985) | <0.001 | 3.403 (1.528, 7.580) | 0.011 |
| TG/HDL-C | Q1 | Reference |  | Reference |  |
|  | Q2 | 1.775 (0.863, 3.653) | 0.113 | 1.534 (0.629, 3.743) | 0.272 |
|  | Q3 | 1.355 (0.641, 2.863) | 0.409 | 1.151 (0.453, 2.924) | 0.715 |
|  | Q4 | 1.855 (1.115, 3.085) | 0.020 | 1.602 (0.846, 3.033) | 0.116 |
| VAI | Q1 | Reference |  | Reference |  |
|  | Q2 | 1.960 (0.989, 3.883) | 0.053 | 1.468 (0.610, 3.531) | 0.312 |
|  | Q3 | 1.921 (1.022, 3.613) | 0.043 | 1.163 (0.500, 2.705) | 0.665 |
|  | Q4 | 2.801 (1.629, 4.818) | <0.001 | 1.563 (0.706, 3.464) | 0.208 |
| CVAI | Q1 | Reference |  | Reference |  |
|  | Q2 | 2.696 (1.110, 6.548) | 0.030 | 2.250 (0.733, 6.911) | 0.122 |
|  | Q3 | 3.052 (1.465, 6.358) | 0.005 | 2.689 (0.928, 7.793) | 0.062 |
|  | Q4 | 4.599 (2.276, 9.294) | <0.001 | 3.988 (1.410, 11.28) | 0.019 |
| LAP | Q1 | Reference |  | Reference |  |
|  | Q2 | 2.179 (0.903, 5.258) | 0.080 | 1.718 (0.542, 5.450) | 0.282 |
|  | Q3 | 2.925 (1.155, 7.408) | 0.026 | 2.134 (0.632, 7.202) | 0.170 |
|  | Q4 | 4.073 (2.016, 8.228) | <0.001 | 2.592 (0.935, 7.187) | 0.062 |

* Adjusted for age, sex, race, marital status, income level, diabetes, hypertension, alcohol frequency, smoking history, glucose-lowering medication taking, and lipid-lowering medication taking.
